# An Efficient Strategy for Small-Scale Screening and Production of Archaeal Membrane Transport Proteins in *Escherichia coli*

**DOI:** 10.1371/journal.pone.0076913

**Published:** 2013-10-07

**Authors:** Pikyee Ma, Filipa Varela, Malgorzata Magoch, Ana Rita Silva, Ana Lúcia Rosário, José Brito, Tânia Filipa Oliveira, Przemyslaw Nogly, Miguel Pessanha, Meike Stelter, Arnulf Kletzin, Peter J. F. Henderson, Margarida Archer

**Affiliations:** 1 Instituto de Tecnologia Quίmica e Biolόgica, Universidade Nova de Lisboa, Oeiras, Portugal; 2 Fachbereich Biologie, Technische Universität Darmstadt, Darmstadt, Germany; 3 School of Biomedical Sciences, Astbury Centre for Structural Molecular Biology, University of Leeds, Leeds, United Kingdom; University of Cambridge, United Kingdom

## Abstract

**Background:**

Membrane proteins play a key role in many fundamental cellular processes such as transport of nutrients, sensing of environmental signals and energy transduction, and account for over 50% of all known drug targets. Despite their importance, structural and functional characterisation of membrane proteins still remains a challenge, partially due to the difficulties in recombinant expression and purification. Therefore the need for development of efficient methods for heterologous production is essential.

**Methodology/Principal Findings:**

Fifteen integral membrane transport proteins from Archaea were selected as test targets, chosen to represent two superfamilies widespread in all organisms known as the Major Facilitator Superfamily (MFS) and the 5-Helix Inverted Repeat Transporter superfamily (5HIRT). These proteins typically have eleven to twelve predicted transmembrane helices and are putative transporters for sugar, metabolite, nucleobase, vitamin or neurotransmitter. They include a wide range of examples from the following families: Metabolite-H^+^-symporter; Sugar Porter; Nucleobase-Cation-Symporter-1; Nucleobase-Cation-Symporter-2; and neurotransmitter-sodium-symporter. Overproduction of transporters was evaluated with three vectors (pTTQ18, pET52b, pWarf) and two *Escherichia coli* strains (BL21 Star and C43 (DE3)). Thirteen transporter genes were successfully expressed; only two did not express in any of the tested vector-strain combinations. Initial trials showed that seven transporters could be purified and six of these yielded quantities of ≥ 0.4 mg per litre suitable for functional and structural studies. Size-exclusion chromatography confirmed that two purified transporters were almost homogeneous while four others were shown to be non-aggregating, indicating that they are ready for up-scale production and crystallisation trials.

**Conclusions/Significance:**

Here, we describe an efficient strategy for heterologous production of membrane transport proteins in *E. coli*. Small-volume cultures (10 mL) produced sufficient amount of proteins to assess their purity and aggregation state. The methods described in this work are simple to implement and can be easily applied to many more membrane proteins.

## Introduction

Up to 30% of the proteins encoded in prokaryotic and eukaryotic genomes are membrane proteins [1]. Malfunctions of some membrane proteins are implicated in various diseases and they are therefore clinically important as potential drug targets [2,3]. Study of membrane proteins is technically challenging and notoriously difficult due to their hydrophobicity and low natural abundance, contributing to the relatively low number of membrane protein (MP) structures deposited in the protein databank (www.rcsb.org, currently around 400 unique MP structures compared to over 90,500 soluble ones). Structural and functional characterisation usually requires milligram quantities of pure protein and optimisation of conditions for the overproduction and purification of membrane proteins is laborious. In the past decade, there have been on-going efforts to develop high-throughput and efficient methods for the production of membrane proteins, however numerous obstacles remain [4–6]. Firstly, due to their low natural abundance, heterologous overexpression of their genes is required and is usually accompanied by toxicity to the host cell. Secondly, the choice of suitable detergents for protein extraction from the cellular membrane is not trivial. Lastly, for X-ray crystallographic studies, the protein sample needs to be pure and homogeneous, as heterogeneity may hamper formation of well-ordered crystals.

To address the obstacles in membrane protein production, we aimed to devise a strategy that will obviate the requirement for moderate to large-scale expression and purification, allowing the screening for multiple potential targets for structural studies. Current therapeutic drugs act on four main types of protein targets, including receptors, enzymes, ion channels and transporters [7]. Our work has focused on transporters. The significance of transport processes in cellular metabolism is emphasised by the identification of over 600 different transport protein families identified through biochemical and genomic studies [8] (www.tcdb.org). In the present work, we describe a workflow enabling heterologous gene expression, purification and a quality test of the produced proteins in small-scale. We selected fifteen archaeal membrane transport proteins from five different families (MHS, SP, NSS, NCS1 and NCS2), which, to our knowledge, have not been previously studied (Table 1). Most of them are homologues to those found in humans that have medical and pharmacological relevance, so Archaea can provide a highly convenient model for elucidating by proxy the molecular mechanisms of mammalian transporters. Transporters from the nucleobase-cation-symporter-1 (NCS1) family have been found to date only in Bacteria, Archaea, yeast, fungi and plants, where they are vital components of salvage pathways for nucleobases, vitamins and related metabolites [8,9]. We have chosen phylogenetically diverse types of Archaea that live in extreme conditions of high temperature, high salt concentration and/or acidic pH.

**Table 1:** List of selected transporter proteins.

**Gene name**	**Uniprot accession**	**Family**	**Putative substrate^a^**	**Organism**	**Size^a^ (kDa)**	**No. of TMs^b^**	**C-term localisation^b^**
SSO2938	Q97UR4	MHS	proline/betaine	*S. solfataricus*	48.6	12	In
SSO2528	Q97VT2	MHS	metabolite	*S. solfataricus*	45.9	12	In
SSO2042	Q97WS4	NCS1	allantoin	*S. solfataricus*	54.5	12	In
SSO1665	Q97XP6	NCS1	cytosine	*S. solfataricus*	48.8	12	In
Saci2039	Q4J795	NCS1	pyrimidine	*S. acidocaldarius*	48.8	11/12	In
MA1518	Q8TQM2	NSS	neurotransmitter	*M. acetivorans*	53.7	12	In
MJ1319	Q58715	NSS	neurotransmitter	*M. jannaschii*	53.5	12	In
Mevan1511	A6USD3	NCS2	uracil/xanthine^c^	*M. vannielii*	43.1	11	In
MMP0681	Q6LZE8	NCS2	uracil/xanthine^c^	*M. maripaludis*	42.9	11	In
Hbor39700	E4NW64	SP	sugar	*H. borinquense*	48.3	12	In
AF2014	Q28265	SP	sugar	*A. fulgidus*	45.0	12	In
Ta0252	Q9HLH6	SP	D-xylose	*T. acidophilum*	52.7	12	In
Msed1117	AYF4S8	SP	sugar	*M. sedula*	50.3	12	In
pNG7043	Q5V6Y0	MHS	metabolite	*H. marismortui*	48.5	12	In
Saci1848	Q4J7S7	MHS	proline/betaine	*S. acidocaldarius*	43.9	12	In

^a^ Putative substrate and theoretical mass from www.uniprot.org. ^b^ Prediction of transmembrane helices and C-terminal localisation by TMHMM [13]. ^c^ Homologues of the human vitamin C transporter. Transmembrane helices (TMs). Inside the cell (In). Metabolite-H^+^-symporter (MHS). Sugar-porter (SP). Nucleobase-cation-symporter 1 or 2 (NCS1 or NCS2). Neurotransmitter-sodium-symporter (NSS). MHS and SP belong to the Major Facilitator Superfamily (MFS). NCS1, NCS2 and NSS belong to the 5-Helix Inverted Repeat Transporter Superfamily (5HIRT).

We amplified genes of 15 transporters from 11 archaeal genomes (Table 1) and sub-cloned each gene into three different expression vectors with a C-terminal poly-histidine affinity tag. Several constructs were also fused to an additional StrepII-tag or green fluorescent protein (GFP). Further diversity was achieved using T7 or tac promoter-driven expression. A collection of 40 constructs was introduced into two different *E. coli* strains and protein production in the membrane fraction was evaluated. Using the described approach, we have successfully produced 13 out of 15 membrane transport proteins, purified seven target proteins, and obtained six non-aggregated proteins (assessed by size-exclusion chromatography; SEC), two of which displayed the desirable SEC profile suitable for structural studies. In summary, we describe here the successful use of the *Escherichia coli* system for overproduction of archaeal membrane transport proteins. The depicted strategy can be easily adapted and used for screening of other membrane proteins.

## Materials and Methods

### Materials

Genomic DNAs were from DSMZ, ATCC or kindly provided by Dr. Sabrina Fröls (Darmstadt, Germany) and Dr. Volker Müller (Frankfurt/Main, Germany). Restriction enzymes were from New England Biolabs (NEB). Isopropyl-β-D-thio-galactoside (IPTG), chloramphenicol and carbenicillin were from ROTH (Karlsruhe, Germany). Kanamycin was from Sigma. n-Dodecyl β-D-maltoside (DDM) was purchased from Affymetrix. The plasmid pTTQ18 used in this work was previously described in Ward et al. [10]. Plasmid pET52b(+) was from Novagen. Plasmid pWarf(-) was kindly provided by Dr. Jeff Abramson (California, Los Angeles). Additional chemicals were from Sigma, unless otherwise stated.

### Target selection and bioinformatics analysis

Archaeal homologues were selected by BLAST searches [11] using the sequences of human transporters of medical importance and the hydantoin transporter of *Microbacterium liquefaciens* (Mhp1) [12] (Figures S1-S5, Table 1). The number of transmembrane helices and C-terminal localisation were predicted using *TMHMM* 2.0 [13]. Putative substrate(s) and masses of the target proteins were extracted from the annotation of UniProtKB (www.uniprot.org). Amino acid sequence alignments were made using Geneious R6.1 created by Biomatters (www.geneious.com) and presented using Jalview [14].

### Construction of expression plasmids

The full-length transporter genes were amplified from genomic DNA using *Pfu* (Fermentas) or *Pwo* (Roche) DNA polymerase with upstream and downstream primers (synthesised by Sigma) listed in Table S1. PCR product was purified using Wizard SV gel and PCR clean-up system (Promega), with or without agarose gel electrophoresis depending on the specificity of the amplified product, and digested with endonucleases corresponding to the restriction sites introduced in the primers. Compatible overhangs were used for some primers when the restriction site(s) for cloning was present in the gene (Table S1). Vectors were digested as indicated in Table S1, purified as above, followed by ligation with the digested genes using T4 DNA ligase (NEB). Chemically competent *E. coli* XL10-Gold cells were used for transformation of the ligation products and colonies obtained subsequently were screened by colony PCR using *Taq* DNA polymerase (NEB). Plasmids were isolated from positive clones using the Wizard Plus SV minipreps DNA purification system (Promega). All constructs were sequenced by Stabvida (Monte da Caparica, Portugal).

### Over-production of archaeal membrane transport proteins

Expression constructs were used to transform two *E. coli* strains, BL21 Star (Invitrogen) and C43(DE3) [15], containing the pRARE2 plasmid. Expression trials were performed as follows. An overnight culture (200 µL) was used to inoculate 10 mL LB medium supplemented with 20 mM glycerol and antibiotics; 34 µg/mL chloramphenicol was added to all cultures to select for pRARE2, 100 µg/mL carbenicillin to select for pTTQ18 and pET52b(+), and 50 µg/mL kanamycin to select for pWarf(-). Cells were cultured at 37°C with shaking (220 rpm). Expression was induced with 0.5 mM IPTG when the optical density at 600 nm reached 0.4-0.8. Cells were harvested 3 hours post-induction and cell pellets were stored at -20°C if membranes were not prepared immediately.

### Preparation of membrane fractions

Total *E. coli* membranes were prepared as described previously by Ward et al. [10] with some modifications. Briefly, the cell pellet was resuspended in 500 µL 0.2 M Tris-HCl pH 8.0 and stirred at room temperature for 20 min. To initiate cell lysis 243 µL of 0.2 M Tris-HCl pH 8.0 containing 1 M sucrose and 1 mM EDTA was first added (t = 0 s), followed by addition of 3.3 µL of 10 mg/mL lysozyme in 0.2 M Tris-HCl pH 8.0 at t = 90 s and 480 µL deionised water at t = 120 s. The sample was mixed at room temperature for 20 min and spheroplasts were sedimented at 20,817 x *g* for 20 min. The pellet was resuspended in 1-1.5 mL deionised water and stirred at room temperature for 30 min. Membranes were then sedimented at 20,817 x *g* for 20 min and resuspended in final buffer (10 mM Tris-HCl pH 8.0 with 5% glycerol and 1 mM mercaptoethanol). An additional step of washing in final buffer was performed for membrane preparations that appeared to be viscous as a result of DNA in the sample. Membranes were stored at -20°C if not analysed or used immediately.

### Analysis of membrane fractions

The protein contents of membrane fractions were estimated by measuring optical density at 280 nm using a Nanodrop spectrophotometer (Thermo Scientific) with a correction factor of 0.375. Readings on the Nanodrop instrument for membrane fractions gave an overestimated value due to the presence of respiratory proteins in the inner membranes, which absorb at 280 nm. 0.375 was determined as the correction factor for protein concentrations in the membrane fraction when measured by the Nanodrop instrument in comparison to those determined by the Schaffner Weissman method [16]. We have implemented this correction factor to obtain an estimate of membranes for loading onto SDS-PAGE and for solubilisation. To assess the amounts of produced target proteins, membranes (~15 µg) were analysed by SDS-PAGE on a 1 mm thick gel, using a BioRad MiniGel setup, with 15% resolving and 4% stacking gels [17], followed by Western blotting using HisProbe-HRP and detection with SuperSignal West Pico chemiluminescent substrate (Thermo Scientific). SDS gels were stained with Coomassie brilliant blue. The amount of target proteins was determined by scanning densitometry.

### Purification of archaeal membrane transport proteins

All purification steps were carried out at 0-4°C using a batch purification method. Total *E. coli* membranes (200 µL) at 1-2 mg/mL were solubilised in 20 mM Tris-HCl pH 8.0, 20 mM imidazole, 300 mM NaCl, 20% v/v glycerol and 0.8% w/v DDM for 1 h. Insoluble proteins were removed by centrifugation at 20,817 x *g* for 20 min. Solubilised proteins were bound to 20 µL Ni-NTA resin (Qiagen) for 30 min, followed by two washes with 20 mM Tris-HCl pH 8.0, 20 mM imidazole, 1 M NaCl, 10% v/v glycerol and 0.02% DDM. For analysis, 10 µl water and 10 µl 4X Laemmli buffer were added directly to the 20 µL resin. Samples of 20 µL were analysed on a 15% SDS-PAGE and Western blotting was performed with HisProbe-HRP. The concentration of purified protein was estimated on the Nanodrop instrument using 1 OD_280nm_ = 1 mg/mL.

### Size-exclusion chromatography of purified proteins

To test the quality of the purified proteins, small-scale purifications were performed as described above but using 300 µl membranes and 30 µL Ni-NTA resins. After the washing steps, His-tagged proteins were eluted with 60 µL of 20 mM Tris-HCl pH 8.0, 500 mM imidazole, 150 mM NaCl, 10% v/v glycerol and 0.02% DDM. Eluted protein was centrifuged at 20,817 x *g* to remove insoluble and precipitated materials. 50 µL of the eluted protein sample after centrifugation was subjected to size exclusion chromatography (SEC) in a Superdex 200 5/150 GL column (GE Healthcare) pre-equilibrated with 20 mM Tris pH 8.0, 300 mM NaCl, 5% glycerol and 0.02% DDM. Fractions of 200 µL were collected. An aliquot (30 µL) taken from each fraction was added to 10 µL 4X Laemmli buffer and analysed on a 15% SDS-PAGE. The gel was then stained using the SilverXpress silver staining kit (Invitrogen).

## Results

### Target selection and generation of constructs

Fifteen integral membrane transport proteins were selected from Archaea (Table 1), microorganisms that live mostly in extreme temperatures, salt and/or pH. The choice was based on the rationale that proteins from these organisms, especially those present in the membrane, are exposed to harsh environments and are likely to be very stable and robust to handle in subsequent studies [18–20]. Moreover, 3D structures from archaeal proteins can provide suitable models for their human counterparts. The selected proteins have calculated molecular masses between 43 and 55 kDa. A search was made for amino acid sequence similarity to human transporters, in particular to proteins from the Major Facilitator Superfamily (MFS) such as the human facilitated glucose transporter (21 to 27% identity, Figure S1) or the human synaptic vesicle proteins (17 to 21% identity, Figure S2), or to the 5-Helix Inverted Repeat Transporter (5HIRT) superfamily, which includes examples such as human neurotransmitter transporters (27 and 29% identity, Figure S3), and to the human vitamin C transporter (27 and 28% identity, Figure S4). The targets also include proteins from the nucleobase-cation-symporter-1 (NCS1) family, showing 19 to 27% sequence identity to the hydantoin transporter of *Microbacterium liquefaciens* (Figure S5) [21,22]. The selected archaeal proteins are secondary active transporters and topology predictions showed that they posses 11 or 12 transmembrane helices with their C-termini in the cytoplasm (Table 1). The latter is important when considering possible fusion of GFP or His-tag to membrane proteins for recombinant protein production, because such fusions were previously reported to work best when located inside the cell [23,24]. Also, our predictions agree with the general observation that approximately 80% of membrane proteins have their C-termini inside the cell and most of them have an even number of transmembrane domains [25,26].

**Figure 1:**
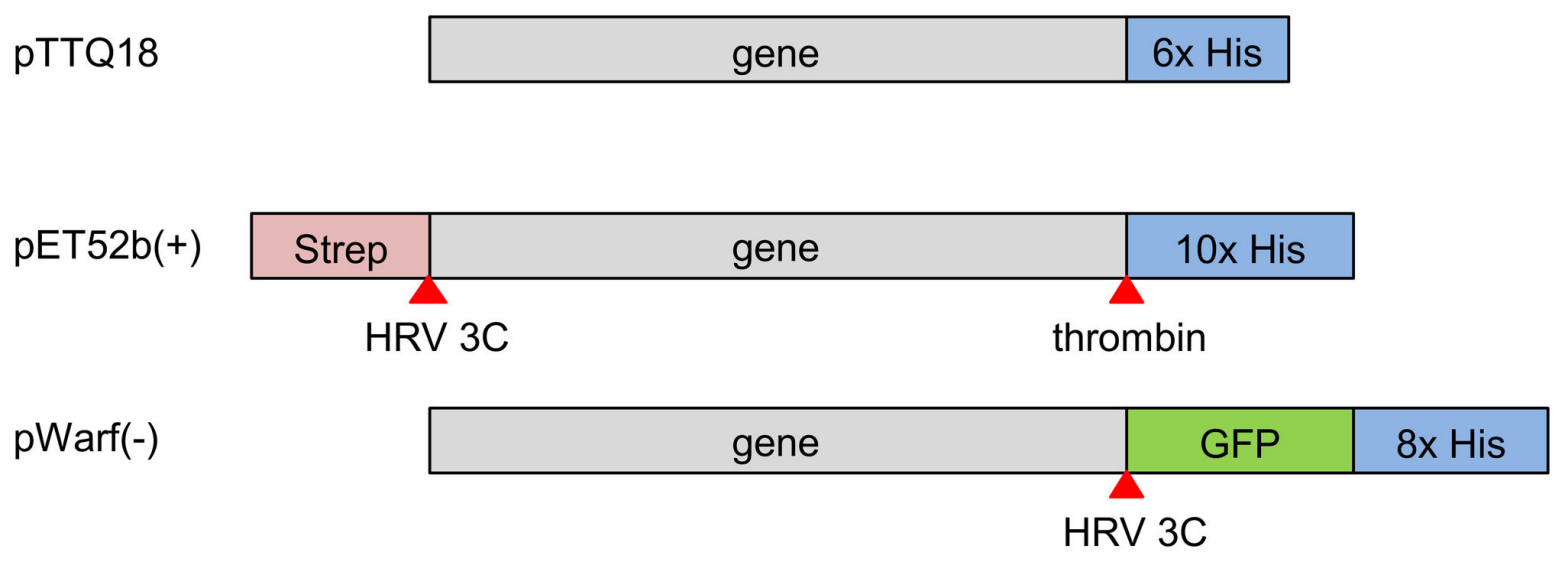
Schematic representation of the protein constructs from overexpression in the different vectors. Additional affinity tags and GFP provided by the vector backbone are colour-coded. Protease cleavage sites are indicated by red arrows.

Three different expression vectors - pTTQ18, pET52b(+) and pWarf(-) were tested. The protein constructs that arise from overexpression in the different vectors are depicted in Figure 1. The pTTQ18 constructs append a C-terminal His-tag. Genes cloned into pET52b(+) introduce an N-terminal StrepII-tag and a C-terminal His-tag in the recombinant proteins; these tags are removable through the presence of flanking HRV 3C and thrombin protease cleavage sites on either side of the target. Proteins produced from the pWarf(-) vector are fused with a C-terminal His-tagged GFP [27], which allows monitoring the target protein by using fluorescent detection [27]. The type of promoter driving gene expression is considered important, especially for membrane proteins. Their production can be toxic to the cells [28,29], so that low levels of expression may actually enhance incorporation into the membrane and improve the quality of difficult target proteins [30]. In these cases, weaker promoters may be preferred for expression of membrane proteins. We have therefore chosen vectors containing either the tac (weaker) or T7 (strong) promoter for comparison. Expression in both the pET52b(+) and pWarf(-) vectors is transcriptionally controlled by the T7 promoter/ *lac* operator [31], whereas in the pTTQ18 vector expression is controlled by the tac promoter, which is more moderate in practice [32,33] and has been previously described in successful production of membrane proteins of up to 4 mg/L in *E. coli* [10,34–36].

In order to maximise the probability of target expression, we utilised a combinatorial approach. Our final collection of clones consisted of 40 constructs representing different versions of 15 transporter genes. In general, the following (with a few exceptions) were constructed for each of the target genes: C-terminal hexa-, octa- or deca-histidine tags; transcriptional control by a T7 or tac promoter; and an additional StrepII tag or GFP fusion.

### Production of thirteen archaeal membrane transport proteins in *E. coli*

**Figure 2:**
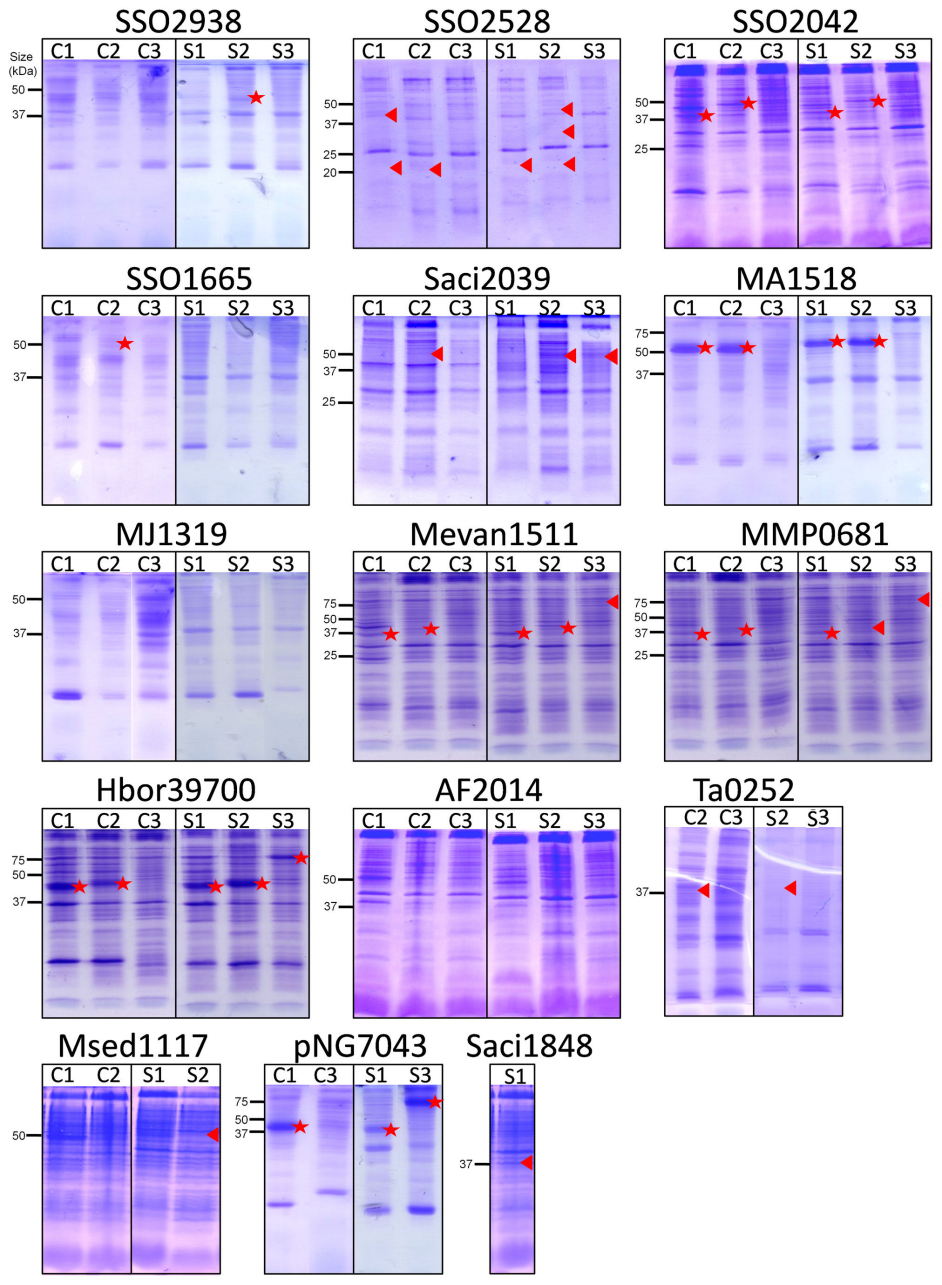
SDS-PAGE analysis of archaeal membrane transport proteins in *E. coli* membranes. Membrane transport protein genes cloned into pTTQ18, pET52b and pWarf were tested in two *E. coli* host strains, C43(DE3) and BL21 Star. Membranes were analysed by SDS-PAGE. C - C43(DE3), S - BL21 Star, 1 - pTTQ18, 2 - pET52b(+), 3 - pWarf(-). Positions of the His-tagged protein determined by Western blotting (see Figure 3) are indicated by: triangle - protein detected by Western blotting but not visible on SDS-PAGE; star - protein detected by Western blotting and visible on SDS-PAGE gels.

**Figure 3:**
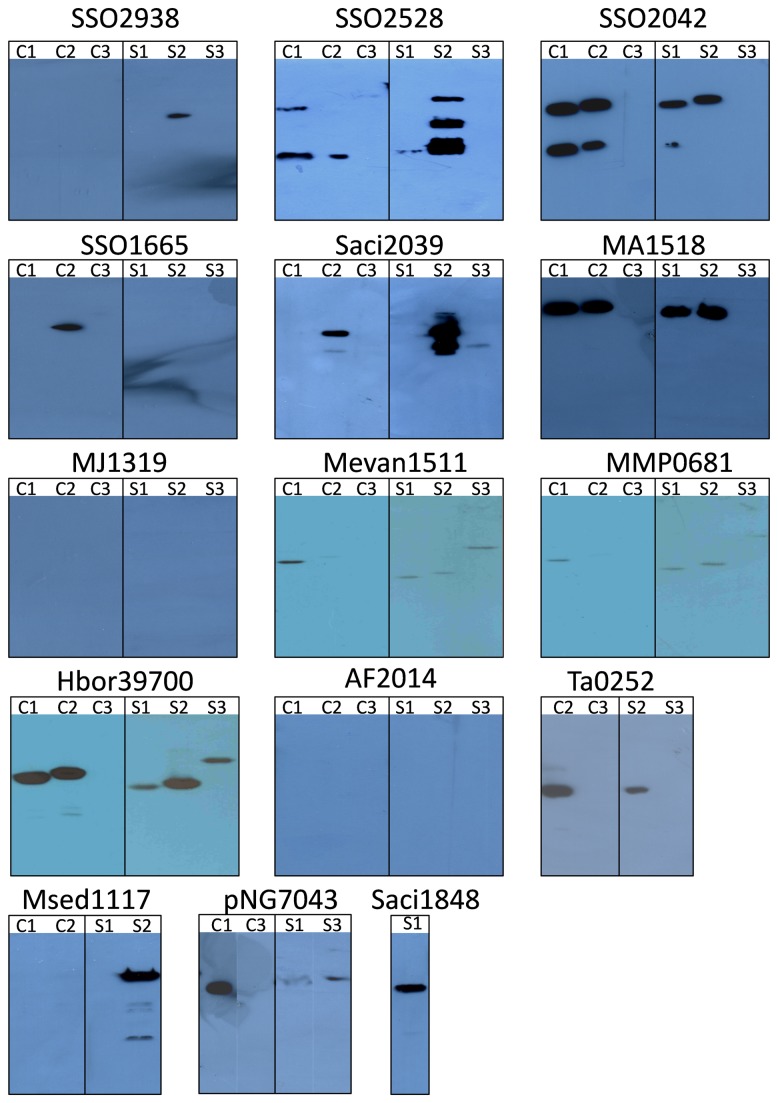
Western blot analysis of archaeal membrane transport proteins in *E. coli* membranes. Membrane transport protein genes cloned into pTTQ18, pET52b and pWarf were tested in two *E. coli* host strains, C43(DE3) and BL21 Star. Membranes were analysed Western blotting. C - C43(DE3), S - BL21 Star, 1 - pTTQ18, 2 - pET52b(+), 3 - pWarf(-).

**Figure 4:**
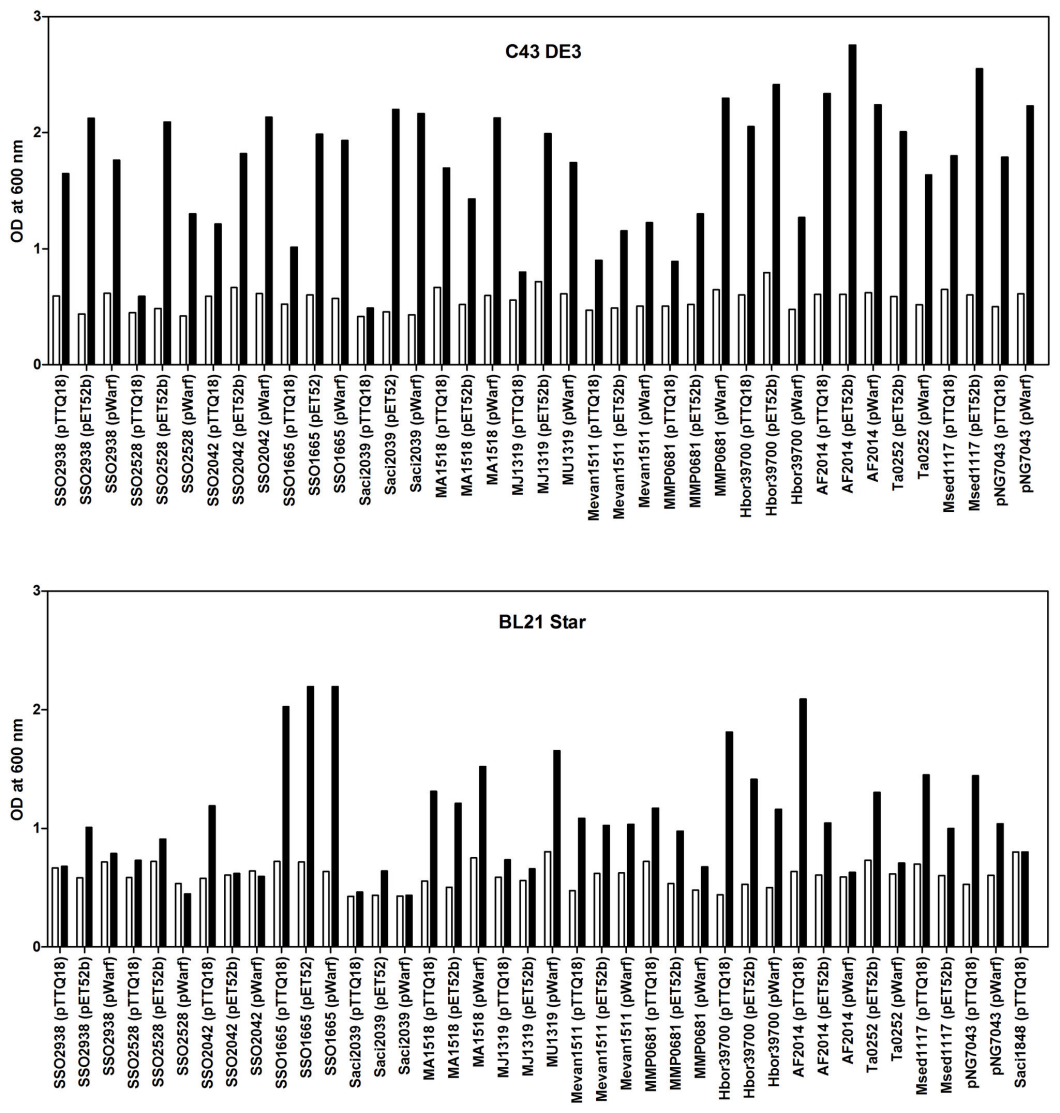
Cell density at induction and harvesting. Expression tests of archaeal transporters were performed in *E. coli* C43(DE3) and BL21 Star strains. Cells were grown in LB medium, induced with 0.5 mM IPTG at OD_600nm_ of 0.4-0.8 and harvested 3 hours post-induction. The bars show the OD_600nm_ at the time of induction (white) and harvesting (black).

*E. coli* is one of the most commonly used expression hosts for production of recombinant proteins because of its short doubling time, achievable high cell densities and inexpensive culture media [37]. We investigated whether different *E. coli* host strains would show significant differences in the expression yield of the target genes. Two *E. coli* strains, C43(DE3) and BL21 Star, were transformed with the target constructs. To compensate for the difference in codon usage between *E. coli* and Archaea, we also transformed the expression strains with the pRARE2 plasmid that encodes seven rare-codon tRNA genes. Cells were grown at 37°C throughout, expression was induced with IPTG at OD_600nm_ of 0.4-0.8 and cells were harvested 3 h after induction. Membranes prepared from small-volume cultures were analysed by SDS-PAGE and Western blotting (Figures 2 and 3). Expression levels are represented as % of the total protein in a membrane preparation and are summarised in Table 2. The harvesting cell densities for C43(DE3) ranged from 0.490 to 2.755 (average OD of 1.720) and 0.435 to 2.190 (average OD of 1.097) for BL21 Star (Figure 4). Cell density was almost two-fold higher when using the C43(DE3) strain, which is one of the Walker strains widely use for membrane protein production to overcome the toxic effects of recombinant proteins, and thus leading to increased yields [15]. However, none of the constructs tested with this host strain showed elevated expression. In fact, pWarf constructs that did not express in this strain were expressed in *E. coli* BL21 Star (Table 2), which is characterised by higher mRNA stability.

**Table 2:** Protein production yields in the different vectors and strains.

	**C43(DE3)**			**BL21 Star**		
**Protein name**	**pTTQ18**	**pET52b(+)**	**pWarf(-)**	**pTTQ18**	**pET52b(+)**	**pWarf(-)**
SSO2938	◊	◊	◊	◊	♦♦ (7%)	◊
SSO2528	♦	♦	◊	♦	♦	◊
SSO2042	♦♦ (2%)	♦♦ (3%)	◊	♦♦ (3%)	♦♦ (3%)	◊
SSO1665	◊	♦	◊	◊	◊	◊
Saci2039	◊	♦	◊	◊	♦	♦
MA1518	♦♦♦ (35%)	♦♦♦ (34%)	◊	♦♦♦ (29%)	♦♦♦ (31%)	◊
MJ1319	◊	◊	◊	◊	◊	◊
Mevan1511	♦♦ (6%)	♦♦ (2%)	◊	♦♦ (5%)	♦♦ (3%)	♦
MMP0681	♦♦ (3%)	♦♦ (2%)	◊	♦♦ (4%)	♦	♦
Hbor39700	♦♦♦ (16%)	♦♦♦ (12%)	◊	♦♦♦ (12%)	♦♦♦ (15%)	♦♦♦ (10%)
AF2014	◊	◊	◊	◊	◊	◊
Ta0252	n.c.	♦	◊	n.c.	♦	◊
Msed1117	◊	◊	n.c.	◊	♦	n.c.
pNG7043	♦♦♦ (30%)	n.c.	◊	♦♦♦ (15%)	n.c.	♦♦♦ (21%)
Saci1848	n.t.	n.c.	n.c.	♦	n.c.	n.c.

◊ no expression (negative on Western blot), ♦ protein detected by Western blotting but not visible on SDS-PAGE, ♦ ♦ protein expressed to 1-10% of total membrane protein, ♦ ♦ ♦ protein expressed to > 10% of total membrane protein. Figures in bracket indicate percentage of expression of target protein in the total membrane determined by scanning densitometry of the SDS-PAGE gels. n.c. = not cloned. n.t. = not tested.

A total of 79 expression tests were performed in both strains of *E. coli* and 39 were positive (49% of tested constructs were detected by Western blotting). The results showed that over half of the tested transporter constructs of pTTQ18 (tac promoter) and pET52b (T7 promoter) were expressed (7/13 of pTTQ18 and 9/13 of pET52b constructs in *E. coli* C43 strain; 8/14 of pTTQ18 and 10/13 of pET52b constructs in *E. coli* Star strain) (Table 2). The majority of the targets that expressed successfully with pTTQ18 also expressed with pET52b showing that the two vectors provide comparable results, with slightly higher success rate in pET52b. Fewer targets were expressed using the pWarf vector; 5/13 of pWarf in BL21 Star, and none in C43 (DE3). Some additional bands of smaller molecular weight than the target protein were present in the SDS-PAGE analysis of membranes from SSO2528, SSO2042, Saci2039 and Msed1117; presumably these represent degradation products or products of incomplete translation.

In summary, 13 out of 15 selected transporter genes were successfully expressed; only two transporters (MJ1319 and AF2014) did not express in any of the vector-strain combinations. At least one construct from each transporter family (MHS, SP, NCS1, NCS2, NSS) was expressed in pTTQ18 and/or pET52b in either host *E. coli* strain. Only a few pWarf constructs expressed in *E. coli* BL21 Star and none in C43(DE3), so it seems likely that the GFP fusion is a burden for production of the selected transport proteins.

### Purification of seven transporters expressed from *E. coli* BL21 Star

Target proteins from membrane preparations that are detected by Western blot but fail to visualise in SDS-PAGE, are usually not the best candidates to pursue for purification using Ni-NTA chromatography because the purified sample will show very low yields and often have contaminants. We decided to proceed with purification using the constructs from *E. coli* BL21 Star, so that we could include targets cloned in all three different vectors. Of the 23 constructs expressed successfully in BL21 Star as judged by Western blotting, 13 were selected for purification because the target proteins were visible in Coomassie Blue stained SDS gels of the membrane preparations (Figure 2 and Figure 3). They covered seven different transporters including Hbor39700 (in pTTQ18, pET52b, pWarf), pNG7043 (in pTTQ18, pWarf), SSO2938 (in pET52b), SSO2042 (in pTTQ18, pET52b), MA1518 (in pTTQ18, pET52b), Mevan1511 (in pTTQ18, pET52b) and MMP0681 (in pTTQ18). For the purification steps, we used dodecyl-maltoside (DDM), since this was previously shown to be one of the most successful detergents both for protein solubilisation and separation in size exclusion chromatography (SEC) for many bacterial, archaeal and eukaryotic membrane proteins [35,38,39].

**Figure 5:**
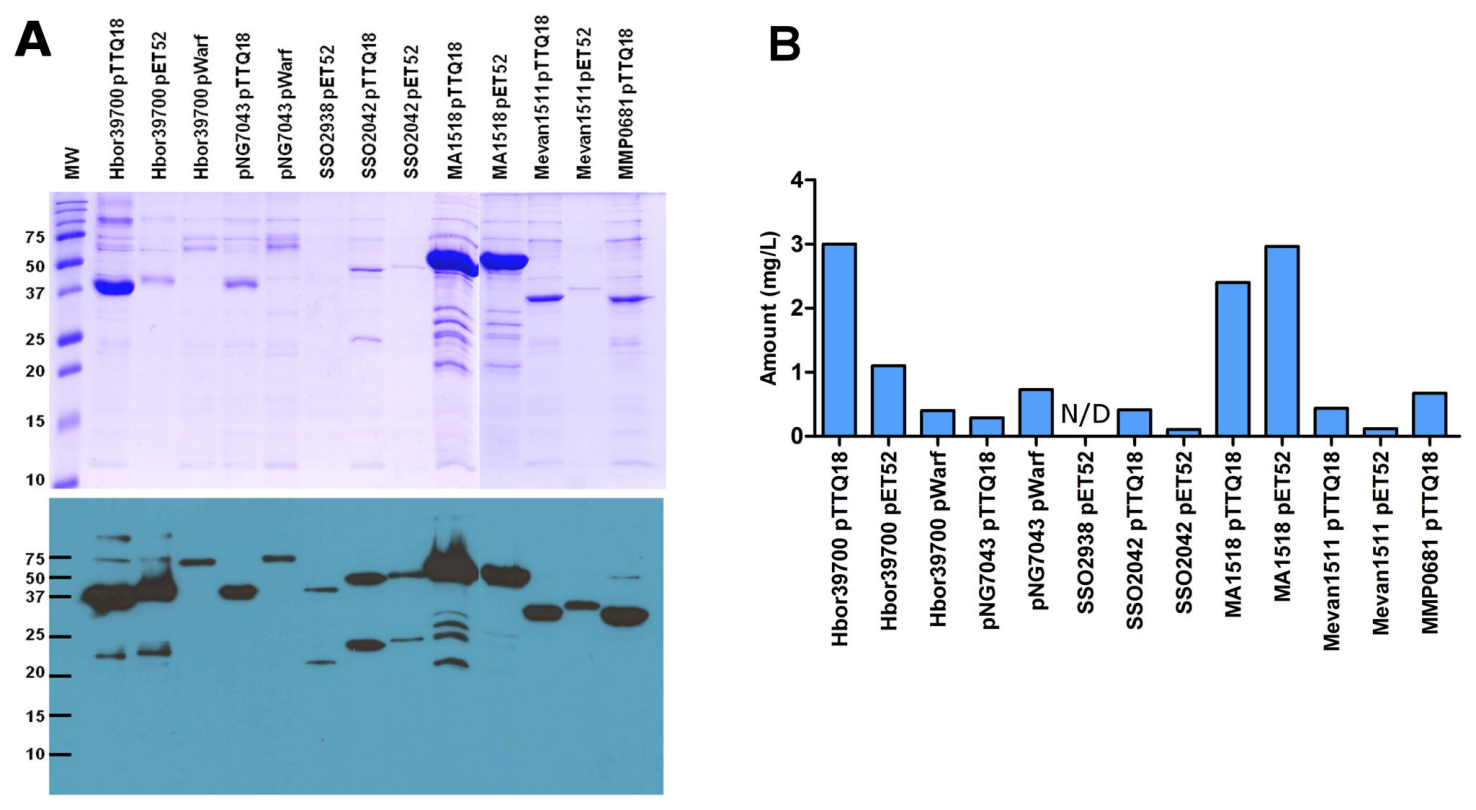
Affinity purification of seven membrane transport proteins. (**A**) Protein purification by Ni-NTA chromatography of 13 constructs expressed in *E. coli* BL21 Star. SDS-PAGE (top panel) and Western blot (bottom panel) of purified protein. MW - Bio-rad precision plus protein standards. (**B**) Estimated protein yield per litre of cell culture after initial Ni-NTA affinity chromatography. N/D - not determined.

A 10 mL culture of *E. coli* BL21 Star harbouring one of the expression vectors yielded approximately 0.5 to 1 mL of membranes at protein concentrations of 1-5 mg/mL. The proteins purified by Ni-NTA chromatography and produced from the different vector constructs carry either a hexa-, octa- or deca-histidine tag (Figure 1). In order to analyze the purity of total bound protein after the washing steps, Ni-NTA resins were added directly to Laemmli buffer and loaded onto SDS gels. Thereby, false negatives were prevented that result from protein precipitation in the resin and no subsequent failure of elution. All of the 13 proteins entered the stacking gel without precipitation or aggregate formation after purification (Figure 5A - stacking gel not shown). Using this one-step procedure, all 13 constructs (representing seven transporters) were purified to 49-97% homogeneity (Figure 5A), as determined by scanning densitometry. The identity of each observed protein band with its respective target was again confirmed by His-tag Western blotting (Figure 5A). Most proteins were intact judged by single bands in SDS-PAGE. Some degradation was observed for Hbor39700 expressed from pTTQ18 and pET52b (purity was 93% and 88% respectively), whereas this effect was not seen when using pWarf. However, the purity from the latter construct was only 55% as a result of a co-purified contaminating protein (Figure 5A). This contaminant was also present in the pNG7043-pWarf protein purification (purity around 49%), indicating that it could be a common contaminant from expression in this vector. Moreover, pNG7043 cloned in the pTTQ18 construct yielded a higher purity of 84%. Although the level of Hbor39700 protein production is very similar when the respective genes were expressed in the three different vectors, the purification yield is higher with the pTTQ18 construct. The reason for this is unclear. However, it is possible that the other two protein constructs are not stable or did not solubilise very well, so further experiments are needed to confirm this. The SS02938-pET52b construct was purified in very low amounts, indicated by a faint band on the SDS-PAGE with some degradation observed in Western blotting (Figure 5A). The SS02042 protein was obviously degraded in the membrane preparation (Figure 2 and Figure 3) and the purified sample also showed two bands; the purity of the protein from the SS02042-pTTQ18 and SSO2042-pET52b constructs was estimated to be 82% and 90%, respectively. Similarly, the purified MA1518 constructs were also degraded, but both pTTQ18 and pET52b constructs yielded similar purities, 94% and 97% respectively. Since degradation products were not seen in their membrane preparations (Figures 2 and 3), it seems that these proteins are susceptible to proteases after extraction from the membrane. Samples from purification of Mevan1511 produced with pTTQ18 and pET52b yielded purities of 77% and 93%, and the MMP0681-pTTQ18 combination yielded 62% purity.

Yields of purified target proteins were estimated to be approximately 0.1-3 mg per litre of culture (Figure 5B). In only the one case of the SSO2938-pET52b construct the yield could not be estimated because the concentration of the eluted protein was too low. In summary, 6 out of the 7 transporters (10/13 constructs) subjected to purification yielded ≥ 0.4 mg/L, sufficient for crystallisation or further studies.

### Assessment of protein stability and homogeneity

The quality of a protein sample needs to be critically assessed for structural studies, as its homogeneity can affect the success of crystallisation. Size-exclusion chromatography (SEC) allows rapid detection of the aggregation state and homogeneity of purified proteins. Homogeneity is a useful indicator of stability as proteins tend to oligomerise or aggregate rapidly when they are unstable. Therefore, we have subjected each of the purified proteins to a homogeneity test using SEC. Proteins were purified by Ni-NTA as described above except that, after the washing steps, proteins were eluted from the resin with 500 mM imidazole. The total eluted proteins per small-scale Ni-NTA purification were calculated to be between 0.6 and 15.6 µg. Due to the low amount of protein loaded onto the SEC column, silver staining was needed for visualisation of the fractions on SDS-PAGE.

**Figure 6:**
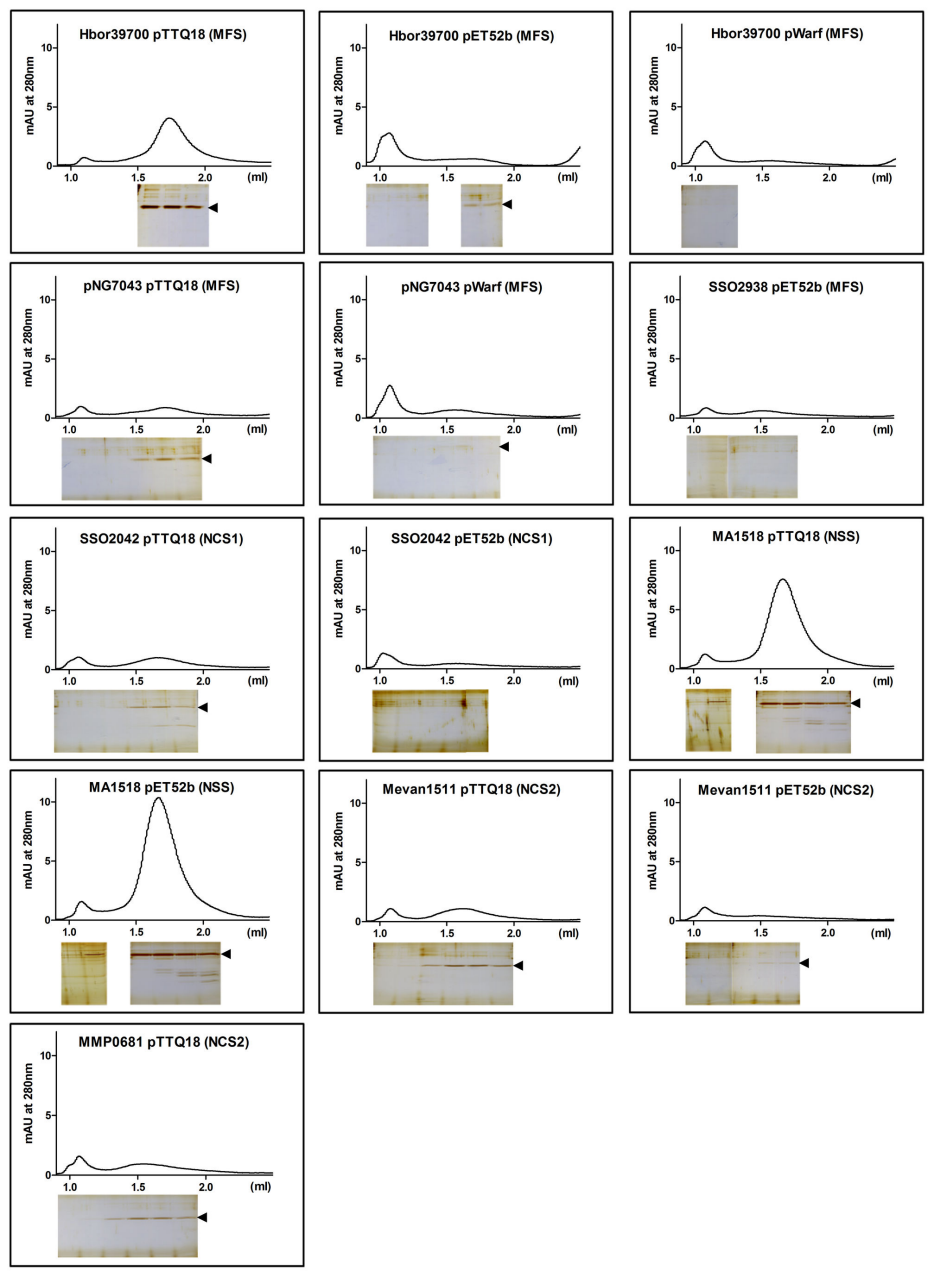
Size-exclusion chromatography of affinity-purified proteins. The affinity-purified proteins were subjected to size-exclusion chromatography on a Superdex 200 5/150 GL column. The elution profile is shown (top panel). The void volume was 1.07 mL determined with Blue dextran. Samples (30 µL) from the elution fractions were analysed on a 15% SDS-PAGE gel stained with SilverXpress silver staining kit (bottom panel). The arrow indicates position of the target protein.

We achieved a high success rate from the homogeneity tests. 6 out of 7 transporters (10/13 constructs) were detected by silver staining after SEC. The majority of these transporters were eluted in the non-void (resolving) volume and appeared to be stable throughout the purification without much aggregation (Figure 6). In particular, for those proteins with a high yield, which includes the two transporters Hbor39700 and MA1518 from three different constructs (Hbor39700-pTTQ18, MA15118-pTTQ18 and MA1518-pET52b), a clear symmetrical peak in the SEC profile appeared, which is an indicator of good quality for subsequent crystallisation studies. The other transporters, also detected by silver staining, were shown to be in the non-aggregated fractions; but because of their low absorbance in the SEC profile their level of heterogeneity is unclear and indicates the need for further optimisation. A peak was observed at the void volume in all SEC profiles. However, in most cases this peak does not show any proteins after silver staining of the electrophoresis gels. It appears to be an artefact, likely due to absorption by the buffers.

## Discussion

Production of integral membrane proteins is time and resource-consuming procedure and often constitutes an obstacle for further studies. The need to use detergents for their extraction from the membranes, to produce sufficient amounts of protein from large-scale cultures, to find the appropriate detergent and buffer conditions to stabilize the MP and yield homogeneous samples, all add to the costs. Finding the optimal conditions frequently requires a trial and error approach. Previous overexpression studies in *E. coli* focused primarily on cell strain, temperature and inducer concentration. More recent studies explored various host systems and protein fusion tags for production of membrane proteins [40–43]. In particular, GFP fusion to membrane proteins facilitates the monitoring or evaluation of the expression levels, purification yields and protein homogeneity by fluorescence detection [27,38,42,44,45]. However, proteolytic removal of the GFP fusion tag may not be trivial and re-cloning of the target into a non-GFP vector may be required [46].

**Figure 7:**
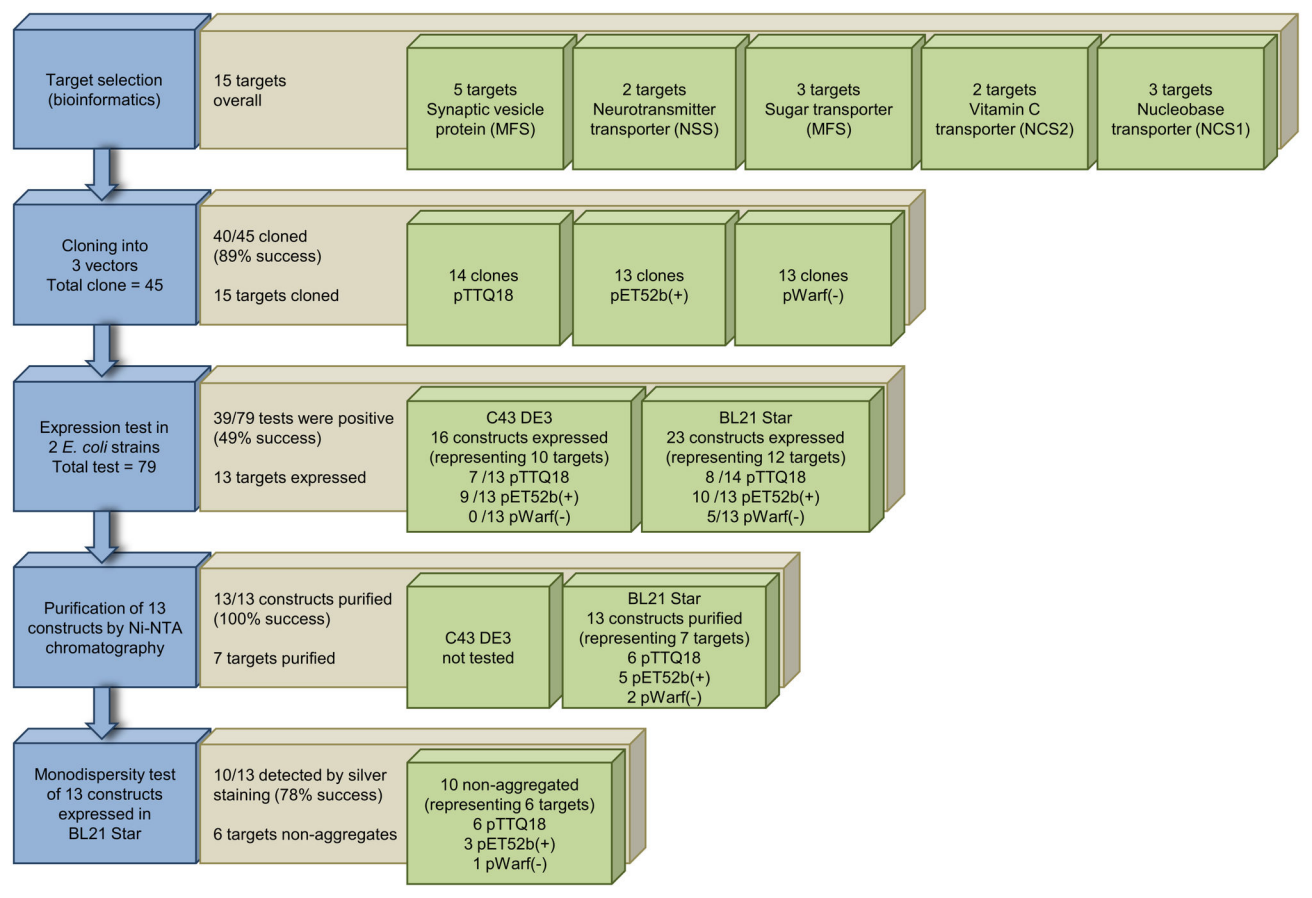
Summary of small-scale production of membrane proteins. Target proteins are selected and cloned into expression vectors. A small-scale expression test is performed, followed by a membrane preparation for purification using Ni-NTA affinity chromatography. Affinity purified proteins were subjected to a homogeneity test. The workflow of (blue), success rates (brown) and results (green) are shown.

Our work focussed on the use of *E. coli* as an expression host highlighting the importance of choosing a suitable vector-strain combination. Our approach offers the advantage of not having to upscale to larger culture volumes in order to purify and further assess the quality of a protein, as compared to most previous studies. We also tried GFP fusion constructs (expression in pWarf), but most of these appeared to be detrimental to the target protein expression and resulted in only a few expressed targets with low yields. In the present study, we describe a method that is applicable for any over-produced membrane protein. The pipeline comprises a minimal number of steps from target selection to assessing a protein’s homogeneity. A summary of the various stages in the presented work is shown in Figure 7.

In summary, 40 constructs (representing 15 target genes) were selected and tests of protein production were performed in *E. coli* C43 and BL21 Star host strains. Overall, 39 of 79 (49%) expression tests were positive. Our results indicate that the choice of vector can affect protein production levels dramatically. A substantially better overproduction of target proteins was observed with pTTQ18 and pET52b constructs (compared to pWarf), and similar expression levels driven by either tac or T7 promoters were obtained. The addition of a GFP fusion appears to impose a burden on the production of target proteins (Figure 2 and Figure 3), which could be due to the increased size of protein construct (an additional 27 kDa from GFP), as it was previously shown that proteins with higher molecular masses were generally more difficult to produce [38,41]. Consistent with previous studies, the pTTQ18 vector confers a good success rate, further extending the use of this vector for over-production of membrane proteins, showing that it is not just limited to bacterial sources but also suitable for the overproduction of archaeal integral membrane transporters [34–36,47]. DDM was a good detergent for extraction of the target proteins from the membrane. Seven different transporters were purified from 13 different vector constructs with DDM as a detergent using single step Ni-NTA chromatography. Proteins were purified to 49-97% with yields of 0.1-3 mg of protein per litre of culture (Figure 5). Since our goal at this stage was to identify promising targets, we did not attempt further optimisation of their purification. Of these 13 proteins, 10 (representing six targets) were present in the non-aggregating fraction, and three (representing two targets) showed the desirable single peak in SEC (Figure 6).

Development of methods for overproduction of membrane proteins in sufficient quantity and quality requires tremendous efforts. Identifying candidates that can be over-produced, solubilised and purified in a stable form for structural and functional studies requires considerable investment in time and resources. The methods described here focus on the production of archaeal membrane transport proteins in *E. coli*. It is the first time that the selected membrane transport proteins were over-produced to significant amounts and purified. The results provide a promising start for further biochemical and structural studies. Moreover, the approach described here does not require robotics and can be performed with minimum expense.

## Supporting Information

Table S1 **Oligonucleotides used in this study.** Primers for cloning of selected target proteins into pTTQ18, pET52b(+) and pWarf(-) vectors. The underlined letters indicate the restriction sites in the primers. Compatible overhangs used for cloning are indicated in bold.
(PDF)

Figure S1 **Alignment of protein sequences of human sugar transporters with archaeal homologues.** Homo sapiens (HS) facilitated glucose transporter member 1 (GLUT1) and member 9 (GLUT9), and fructose transporter aligned with the archaeal homologues from the MFS superfamily.
(TIF)

Figure S2 **Alignment of protein sequences of human synaptic vesicle proteins with archaeal homologues.** Synaptic vesicle protein isoform CRA_c (SV2_isof_CRA_c) and its paralogue synaptic vesicle protein-like isoform 1 (SVOPL_isof_1) from Homo sapiens (HS) were aligned with the archaeal homologues from the MFS superfamily.
(TIF)

Figure S3 **Alignment of protein sequences of human neurotransmitter transporter with archaeal homologues.** Homo sapiens (HS) dopamine, noradrenaline, creatine, GABA and proline transporters were aligned with archaeal homologues of the NSS family.
(TIF)

Figure S4 **Alignment of protein sequences of human vitamin C transporter with archaeal homologues.** Homo sapiens (HS) vitamin C transporter 1 (VCT1) and 2 (VCT2) aligned with archaeal homologues of the NCS2 family. Similarity using score matrix.
(TIF)

Figure S5 **Alignment of protein sequences of *Microbacterium liquefaciens* hydantoin transporter with archaeal homologues.** Hydantoin transporter from *M. liquefaciens* (Mhp1) aligned with archaeal transporters of the NCS1 family.
(TIF)
