# Correction: An Efficient Strategy for Small-Scale Screening and Production of Archaeal Membrane Transport Proteins in *Escherichia coli*

**DOI:** 10.1371/annotation/ba41a7db-2c22-4ffc-b603-526534594a51

**Published:** 2013-11-12

**Authors:** Pikyee Ma, Filipa Varela, Malgorzata Magoch, Ana Rita Silva, Ana Lúcia Rosário, José Brito, Tânia Filipa Oliveira, Przemyslaw Nogly, Miguel Pessanha, Meike Stelter, Arnulf Kletzin, Peter J. F. Henderson, Margarida Archer

An affiliation of the ninth author is missing from the byline. In addition to institution number 1, Miguel Pessanha is also affiliated with the following institution: Escola Superior de Saúde - Instituto Politécnico da Guarda, Guarda, Centro, Portugal.

In addition, a grant from the Fundação para a Ciência e a Tecnologia to the ninth author was incorrectly omitted from the Funding Statement. The grant number is: SFRH/BPD/43202/2008. 

