# Supplementary material for: An Efficient Strategy for Small-Scale Screening and Production of Archaeal Membrane Transport Proteins in Escherichia coli
Source: PLoS One. 2013 Oct 7;8(10):e76913. doi: 10.1371/journal.pone.0076913 (PMC3838208; doi:10.1371/journal.pone.0076913)
Supplement: Table S1 — Oligonucleotides used in this study. Primers for cloning of selected target proteins into pTTQ18, pET52b(+) and pWarf(-) vectors. The underlined letters indicate the restriction sites in the primers. Compatible overhangs used for cloning are indicated in bold. (PDF) [file pone.0076913.s001.pdf]

Gene (Family)	Vector	Restriction digestion of vector	**Primers 5' to 3'**<br>**Downstream and upstream (top and bottom)**
SSO2938 (MFS)	pTTQ18	*Nde*I, *Pst*I	CCGGAATTCGCATATGGTCTCAGAGAAAACTAAG<br>AAAACTGCAGCATATTCCTGCTTCAGTATTTCTTCTCCTT
	pET52b(+)	*Xma*I, *Sac*I	TCCCCCCGGGATGGTCTCAGAGAAAACTAAGGCTATAAT<br>TATGAGCTCATATTCCTGCTTCAGTATTTCTT
	pWarf(-)	*Xho*I, *Bam*HI	CCGCTCGAGATGGTCTCAGAGAAAACTAAGGCTATAAT<br>CGCGGATCCATATTCCTGCTTCAGTATTTCTT
SSO2528 (MFS)	pTTQ18	*Nde*I, *Pst*I	GGGAATTCCATATGAAAGATGAAACTAAAGTTTCCATAGC<br>CCA**ATGCAT**CCCTTACTCTTTCCTGCCCTTCAAATA
	pET52b(+)	*Xma*I, *Sac*I	TCCCCCCGGGATGAAAGATGAAACTAAAGTTTCCATAGC<br>TATGAGCTCCCTTACTCTTTCCTGCCCTTCAAAT
	pWarf(-)	*Xho*I, *Bam*HI	CCGCTCGAGATGAAAGATGAAACTAAAGTTTCC<br>CGCGGATCCCCTTACTCTTTCCTGCCCTTCAAATA
SSO2042 (NCS1)	pTTQ18	*Eco*RI, *Pst*I	CCG**CAATTG**GCATATGTCAGAACAAGAAATAAACCAC<br>AAAACTGCAGCAGGTTCCATTTTCACTTCTC
	pET52b(+)	*Xma*I, *Sac*I	TCCCCCCGGGATGTCAGAACAAGAAATAAACCAC<br>TATGAGCTCAGGTTCCATTTTCACTTCTCTTTT
	pWarf(-)	*Xho*I, *Bam*HI	CCGCTCGAGATGTCAGAACAAGAAATAAACCACGTTAAT<br>CGCGGATCCAGGTTCCATTTTCACTTCTCTTTT
SSO1665 (NCS1)	pTTQ18	*Eco*RI, *Pst*I	CCG**CAATTG**GCATATGACTGGAAAGGAGGAAATTAGCTCAAAATACG<br>CCA**ATGCAT**CTCTTAATATCCCCCCTTCATAGTTAAACG
	pET52b(+)	*Xma*I, *Sac*I	TCCCCCCGGGATGACTGGAAAGGAGGAAATTAGCTCAAAATA<br>TATGAGCTCTCTTAATATCCCCCCTTCATAGTTAAACG
	pWarf(-)	*Xho*I, *Bam*HI	CCGCTCGAGATGACTGGAAAGGAGGAAATTAGCTCAAAATA<br>CGCGGATCCTCTTAATATCCCCCCTTCATAGTTAAA
Saci2039 (NCS1)	pTTQ18	*Eco*RI, *Pst*I	CCGGAATTCGCATATGGAAGGAAAAAAAAGTAAAGA<br>AAAACTGCAGCTGATAATCTCCCACCTTTATAAGTGTA
	pET52b(+)	*Xma*I, *Sac*I	TCCCCCCGGGATGGAAGGAAAAAAAAGTAAAGAGG<br>TATGAGCTCTGATAATCTCCCACCTTTATAAG
	pWarf(-)	*Xho*I, *Bam*HI	CCGCTCGAGATGGAAGGAAAAAAAAGTAAAG<br>CGCGGATCCTGATAATCTCCCACCTTTATAAGTGTA
MA1518 (NSS)	pTTQ18	*Eco*RI, *Pst*I	CCG**CAATTG**GCATATGGTGGCGAGAGAAGTCTGGAACA<br>CCA**ATGCAT**CCTTCACCTCCCTGTTGATAAAAGGC
	pET52b(+)	*Bam*HI, *Sac*I	CGCGGATCCGATGGTGGCGAGAGAAGTCTGGAACACCAGAGT<br>TATGAGCTCCTTCACCTCCCTGTTGATAAAAGGCGTAAGGAT
	pWarf(-)	*Xho*I, *Bam*HI	CCGCTCGAGATGGTGGCGAGAGAAGTCTGGAACA<br>CGCGGATCCCTTCACCTCCCTGTTGATAAAAG
MJ1319 (NSS)	pTTQ18	*Eco*RI, *Pst*I	CCGGAATTCGCATATGAGTTATATGGAAAGAGAAAGCTG<br>AAAACTGCAGCCCAACCTTTAATCGTTTTCAT
	pET52b(+)	*Xma*I, *Not*I	TCCCCCCGGGATGAGTTATATGGAAAGAGAAAGCTG<br>AAGGAAAAAAGCGGCCGCCCAACCTTTAATCGTTTTCAT
	pWarf(-)	*Xho*I, *Bam*HI	CCGCTCGAGATGAGTTATATGGAAAGAGAAAGCTG<br>CGCGGATCCCCAACCTTTAATCGTTTTCATTT
Mevan1511 (NCS2)	pTTQ18	*Eco*RI, *Pst*I	CCGGAATTCGCATATGAAAAGAATAGTATTGGGTTTTCAA<br>CCA**ATGCAT**CTTTTTTTAATATCTGATCCA
	pET52b(+)	*Xma*I, *Sac*I	TCCCCCCGGGATGAAAAGAATAGTATTGGGTTTTCAAC<br>TATGAGCTCTTTTTTTAATATCTGATCCATCAATATTGC
	pWarf(-)	*Xho*I, *Bam*HI	CCGCTCGAGATGAAAAGAATAGTATTGGGTT<br>CGCGGATCCCCTCAACACCTGCTCTAACACAATA
MMP0681 (NCS2)	pTTQ18	*Eco*RI, *Pst*I	CCGGAATTCGCATATGAAAAGAATAGCACTAGGTTTCCA<br>CCA**ATGCAT**CTTTTTTTAACAACTGGTCCATTAATATTGCG
	pET52b(+)	*Xma*I, *Sac*I	TCCCCCCGGGATGAAAAGAATAGCACTAGGTTTCCAA<br>TATGAGCTCTTTTTTTAACAACTGGTCCATT
	pWarf(-)	*Xho*I, *Bam*HI	CCGCTCGAGATGAAAAGAATAGCACTAGGTTTCC<br>CGCGGATCCTTTTTTTAACAACTGGTCCATT
Hbor39700 (MFS)	pTTQ18	*Nde*I, *Pst*I	GGGAATTCCATATGTCAACCTCTAATATACAGGACATTCT<br>CCA**ATGCAT**CGTCGTCGGCGGTCGATGCGATATTCTT
	pET52b(+)	*Xma*I, *Sac*I	TCCCCCCGGGATGTCAACCTCTAATATACAGGACATTC<br>TATGAGCTCGTCGTCGGCGGTCGATGCGATATTC
	pWarf(-)	*Eco*RI, *Bam*HI	CCG**CAATTG**CCATGTCAACCTCTAATATACAGGACATTCT<br>CGCGGATCCGTCGTCGGCGGTCGATGCGATATTC

AF2014 (MFS)	pTTQ18	*Nde*I, *Pst*I	GGGAATTCCATATGGGTGAATTCTCCAAGTTTGAGAAAC<br>AAAACTGCAGCAAACTCCTCCAGCTCTCTTCCCTT
	pET52b(+)	*Xma*I, *Sac*I	TCCCCCCGGGATGGGTGAATTCTCCAAGTTTGAGAAACTA<br>TATGAGCTCAAACTCCTCCAGCTCTCTTCCCTTCGTTTC
	pWarf(-)	*Xho*I, *Bam*HI	CCGCTCGAGATGGGTGAATTCTCCAAGTTTGAGAAACTA<br>CGCGGATCCAAACTCCTCCAGCTCTCTTCCCTTCGTTTC
Ta0252 (MFS)	pTTQ18	*Eco*RI, *Pst*I	CCGGAATTCGCATATGGAAAATGGAGTCAGATATTCCGAT<br>AAAACTGCAGCTTTCGGGTCTGCTGGAACGCTCTCCTT
	pET52b(+)	*Xma*I, *Sac*I	TCCCCCCGGGATGGAAAATGGAGTCAGATATTCCGAT<br>TATGAGCTCTTTCGGGTCTGCTGGAACGCTCTCCTT
	pET52b(+)	*Xho*I, *Bam*HI	CCGCTCGAGATGGAAAATGGAGTCAGATATTCCG<br>CGCGGATCCTTTCGGGTCTGCTGGAACGCTCTCCTTCTT
Msed1117 (MFS)	pTTQ18	*Eco*RI, *Pst*I	CCGGAATTCGCATATGGTTATCAAGAGTAATCCAGACAAGG<br>AAAACTGCAGCTCTAAATCTATCAGTTATTTCTTCTA
	pET52b(+)	*Xma*I, *Sac*I	TCCCCCCGGGATGGTTATCAAGAGTAATCCAGACAA<br>TATGAGCTCTCTAAATCTATCAGTTATTTCT
	pWarf(-)	*Xho*I, *Bam*HI	CCGCTCGAGATGGTTATCAAGAGTAATCCAGACAA<br>CGCGGATCCTCTAAATCTATCAGTTATTTCTT
pNG7043 (MFS)	pTTQ18	*Eco*RI, *Hind*III	CCGGAATTCGCATATGTCCACAGCGACCATACGTAAC<br>CCCAAGCTTCTATTAATGGTGATGGTGATGGTGGCTGCCACGGTCAGCCAGTGAGATGTTCTGC
	pET52b(+)	*Bam*HI, *Sac*I	CGCGGATCCGATGTCCACAGCGACCATACGTAAC<br>TATGAGCTCGTCAGCCAGTGAGATGTTCTGC
	pWarf(-)	*Eco*RI, *Bam*HI	CCGGAATTCCCATGTCCACAGCGACCATACGTAA<br>CGCGGATCCGTCAGCCAGTGAGATGTTCTGCCGGAGGTC
Saci1848 (MFS)	pTTQ18	*Eco*RI, *Pst*I	CCGGAATTCGCATATGAGGAACAATACCACTAAC<br>AAAACTGCAGCTATTTTCTCTAAATTCACATTAT
	pET52b(+)	*Xma*I, *Sac*I	TCCCCCCGGGATGAGGAACAATACCACTAACG<br>TATGAGCTCTATTTTCTCTAAATTCACATTATTAGT
	pWarf(-)	*Xho*I, *Bam*HI	CCGCTCGAGATGAGGAACAATACCACTAACGTGAT<br>CGCGGATCCTATTTTCTCTAAATTCACATTATTAGT
