# Supplementary figures and images for: An Efficient Strategy for Small-Scale Screening and Production of Archaeal Membrane Transport Proteins in Escherichia coli
Source: PLoS One. 2013 Oct 7;8(10):e76913. doi: 10.1371/journal.pone.0076913 (PMC3838208; doi:10.1371/journal.pone.0076913)

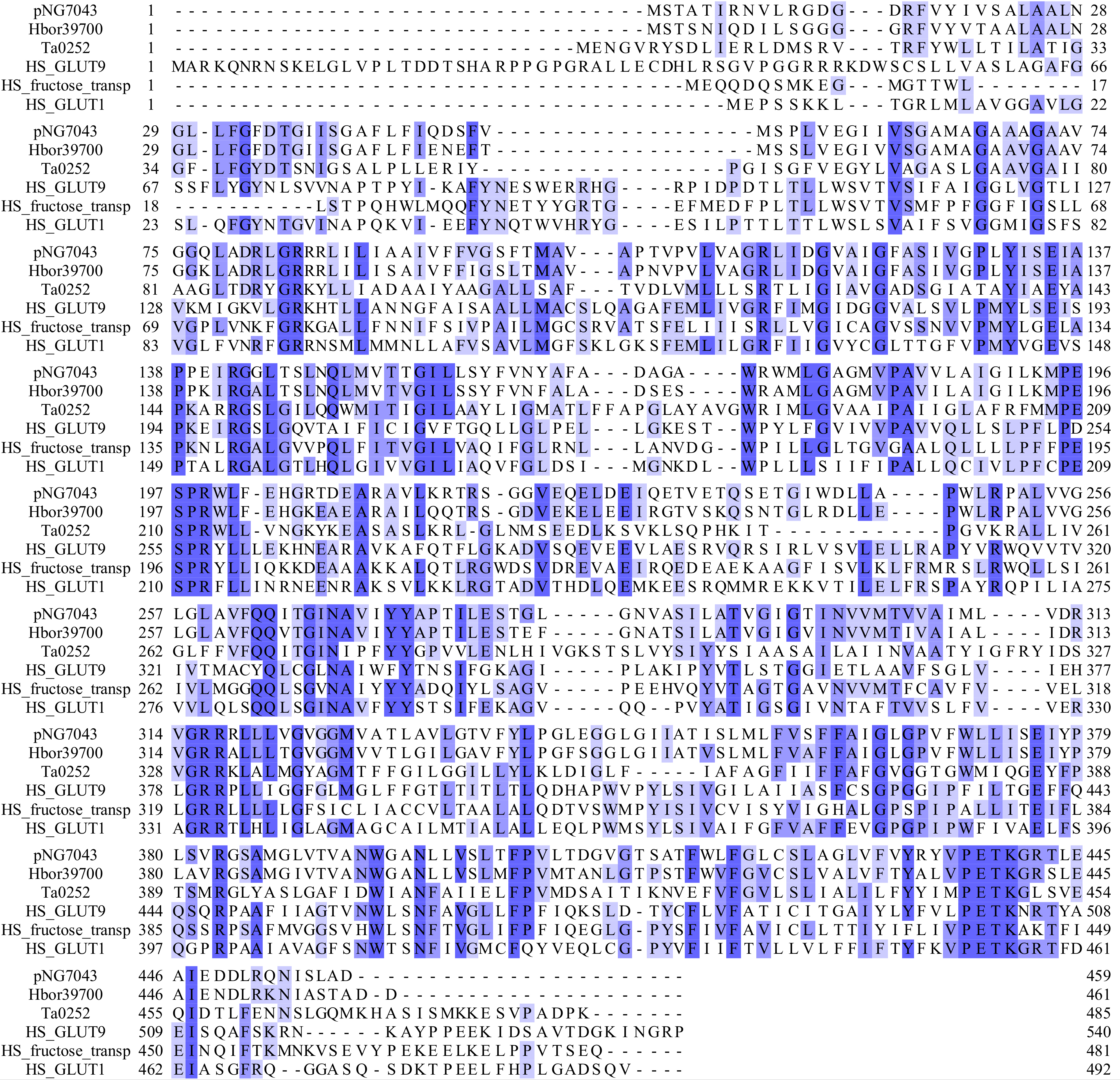

Supplement: Figure S1 — Alignment of protein sequences of human sugar transporters with archaeal homologues. Homo sapiens (HS) facilitated glucose transporter member 1 (GLUT1) and member 9 (GLUT9), and fructose transporter aligned with the archaeal homologues from the MFS superfamily. (TIF) [file pone.0076913.s002.tif]

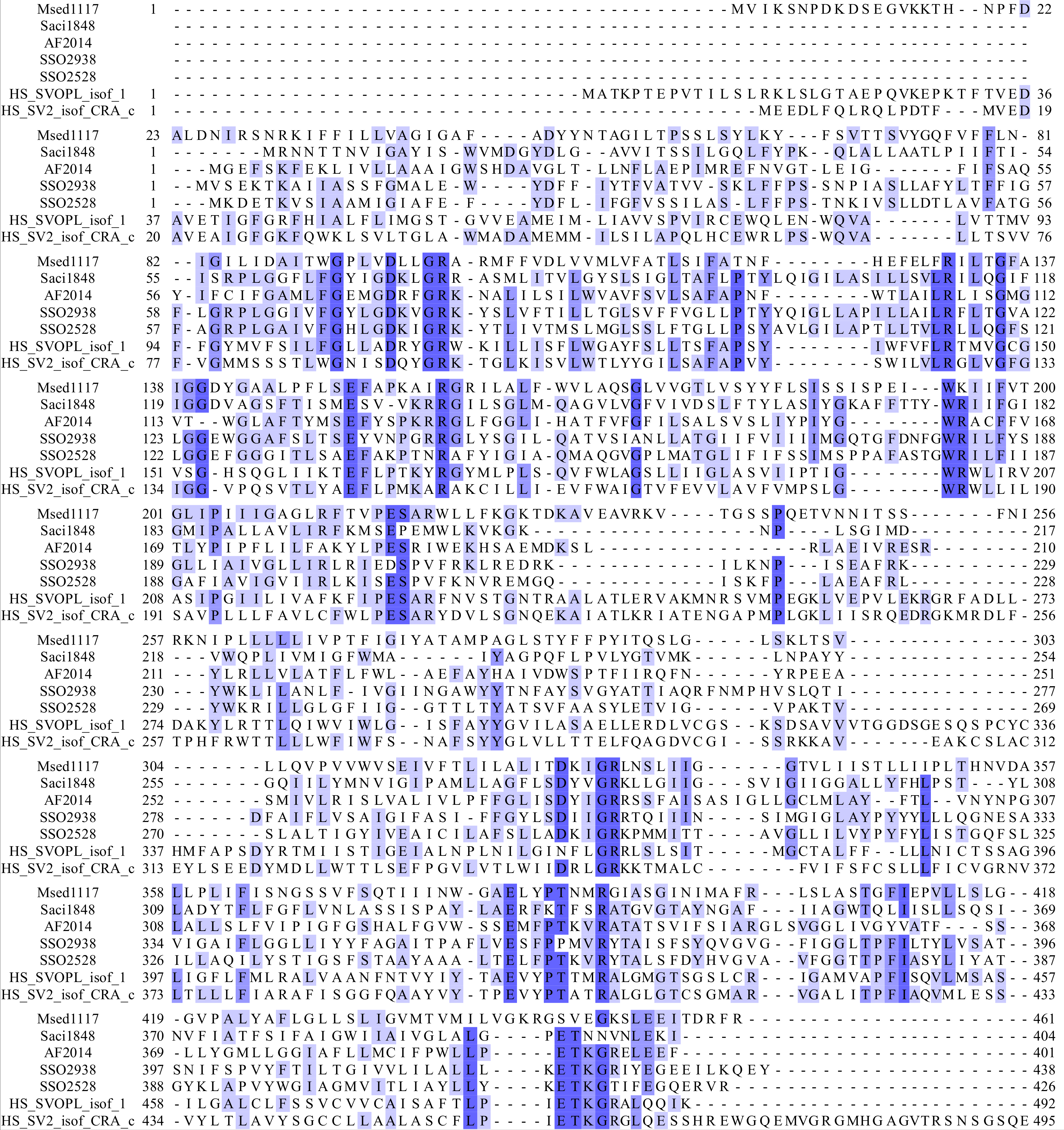

Supplement: Figure S2 — Alignment of protein sequences of human synaptic vesicle proteins with archaeal homologues. Synaptic vesicle protein isoform CRA_c (SV2_isof_CRA_c) and its paralogue synaptic vesicle protein-like isoform 1 (SVOPL_isof_1) from Homo sapiens (HS) were aligned with the archaeal homologues from the MFS superfamily. (TIF) [file pone.0076913.s003.tif]

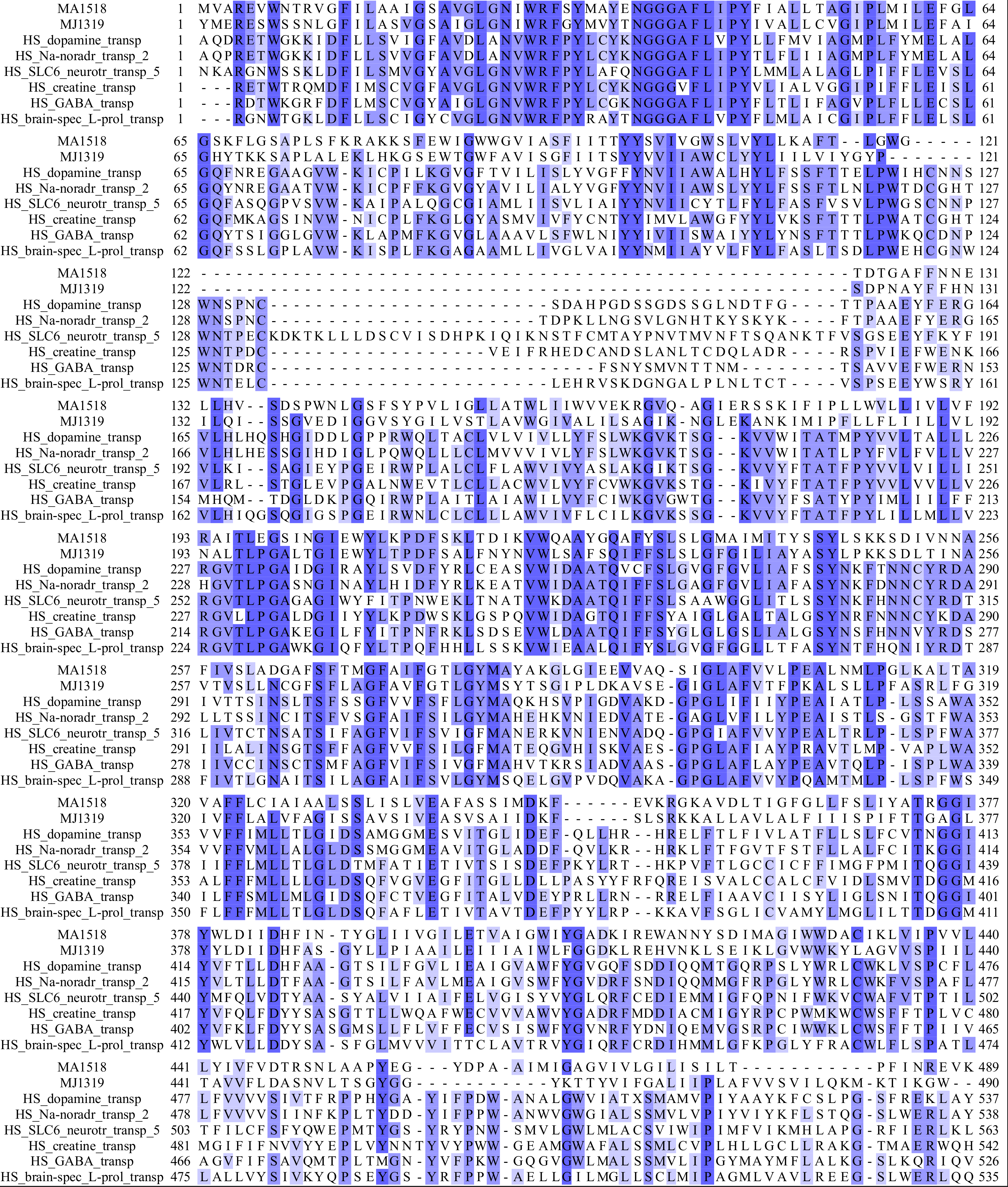

Supplement: Figure S3 — Alignment of protein sequences of human neurotransmitter transporter with archaeal homologues. Homo sapiens (HS) dopamine, noradrenaline, creatine, GABA and proline transporters were aligned with archaeal homologues of the NSS family. (TIF) [file pone.0076913.s004.tif]

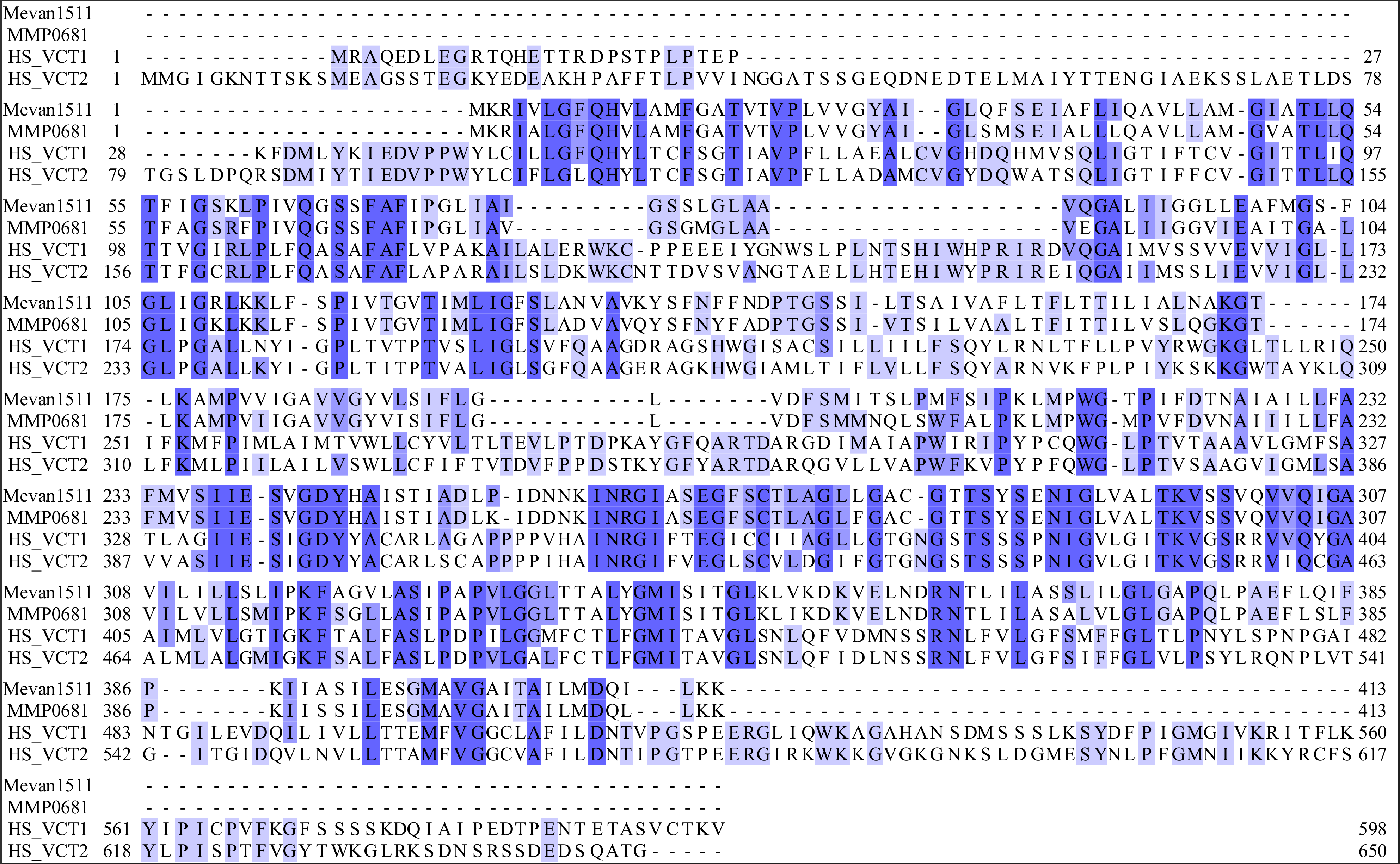

Supplement: Figure S4 — Alignment of protein sequences of human vitamin C transporter with archaeal homologues. Homo sapiens (HS) vitamin C transporter 1 (VCT1) and 2 (VCT2) aligned with archaeal homologues of the NCS2 family. Similarity using score matrix. (TIF) [file pone.0076913.s005.tif]

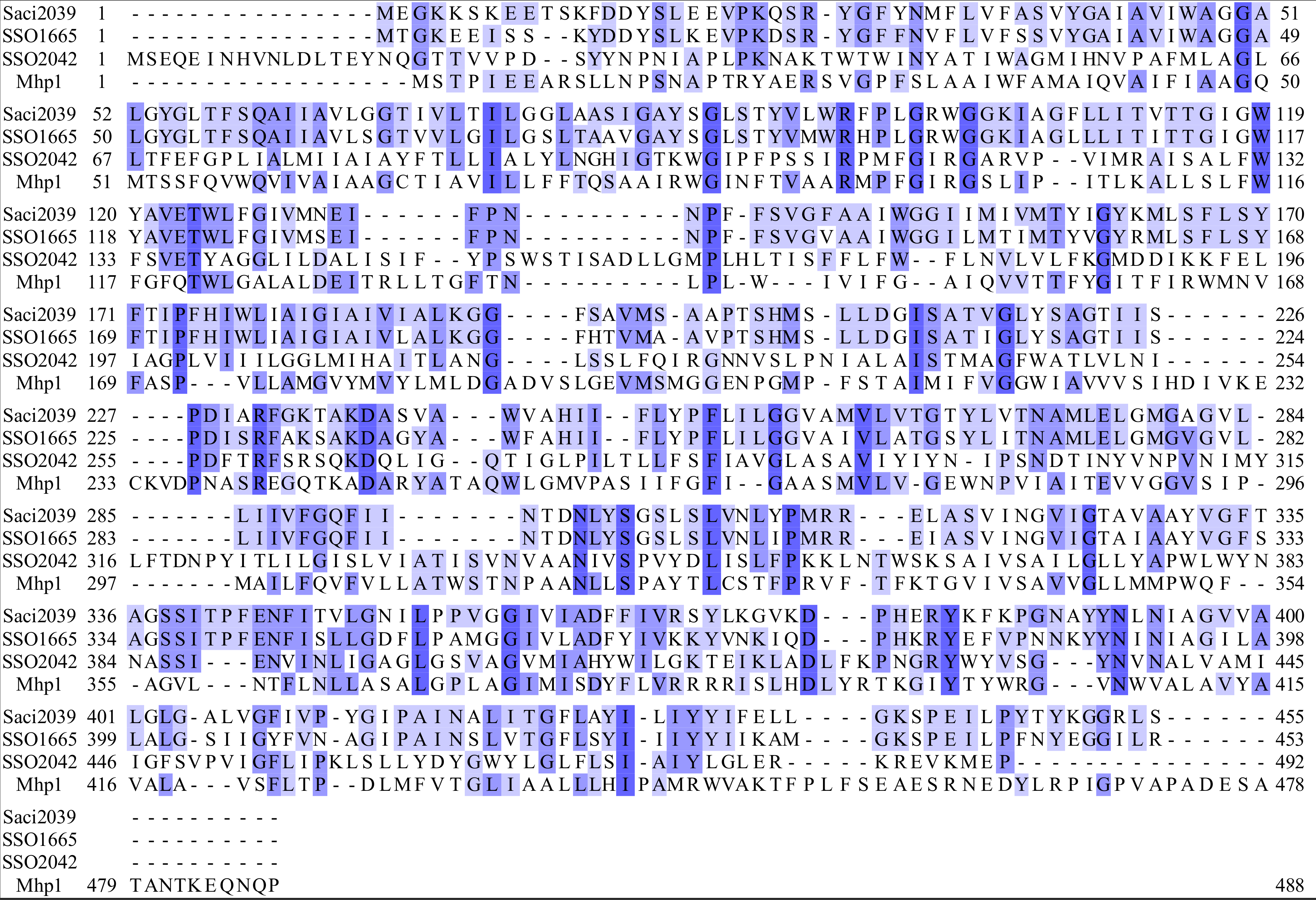

Supplement: Figure S5 — Alignment of protein sequences of Microbacterium liquefaciens hydantoin transporter with archaeal homologues. Hydantoin transporter from M. liquefaciens (Mhp1) aligned with archaeal transporters of the NCS1 family. (TIF) [file pone.0076913.s006.tif]
